# PRC2 mediated KLF2 down regulation: a therapeutic and diagnostic axis during tumor progression

**DOI:** 10.1186/s12935-023-03086-3

**Published:** 2023-10-08

**Authors:** Negin Taghehchian, Amirhosein Maharati, Iman Akhlaghipour, Amir Sadra Zangouei, Meysam Moghbeli

**Affiliations:** 1 Medical Genetics Research Center, Mashhad University of Medical Sciences, Mashhad, Iran; 2 Student Research Committee, Faculty of Medicine, Mashhad University of Medical Sciences, Mashhad, Iran; 3 Department of Medical Genetics and Molecular Medicine, School of Medicine, Mashhad University of Medical Sciences, Mashhad, Iran

**Keywords:** KLF2, Cancer, Polycomb complex, Tumor suppressor, Diagnosis, Marker, Targeted therapy

## Abstract

Surgery and chemo-radiotherapy are used as the common first-line treatment options in many cancers. However, tumor relapse is observed in many cancer patients following such first-line treatments. Therefore, targeted therapy according to the molecular cancer biology can be very important in reducing tumor recurrence. In this regard, a wide range of monoclonal antibodies against the growth factors and their receptors can offer more targeted treatment in cancer patients. However, due to the importance of growth factors in the normal biology of body cells, side effects can also be observed following the application of growth factor inhibitors. Therefore, more specific factors should be introduced as therapeutic targets with less side effects. Krüppel-like factors 2 (KLF2) belongs to the KLF family of transcription factors that are involved in the regulation of many cellular processes. KLF2 deregulations have been also reported during the progression of many tumors. In the present review we discussed the molecular mechanisms of KLF2 during tumor growth and invasion. It has been shown that the KLF2 as a tumor suppressor is mainly inhibited by the non-coding RNAs (ncRNAs) through the polycomb repressive complex 2 (PRC2) recruitment. This review is an effective step towards introducing the KLF2 as a suitable diagnostic and therapeutic target in cancer patients.

## Background

**Fig. 1:**
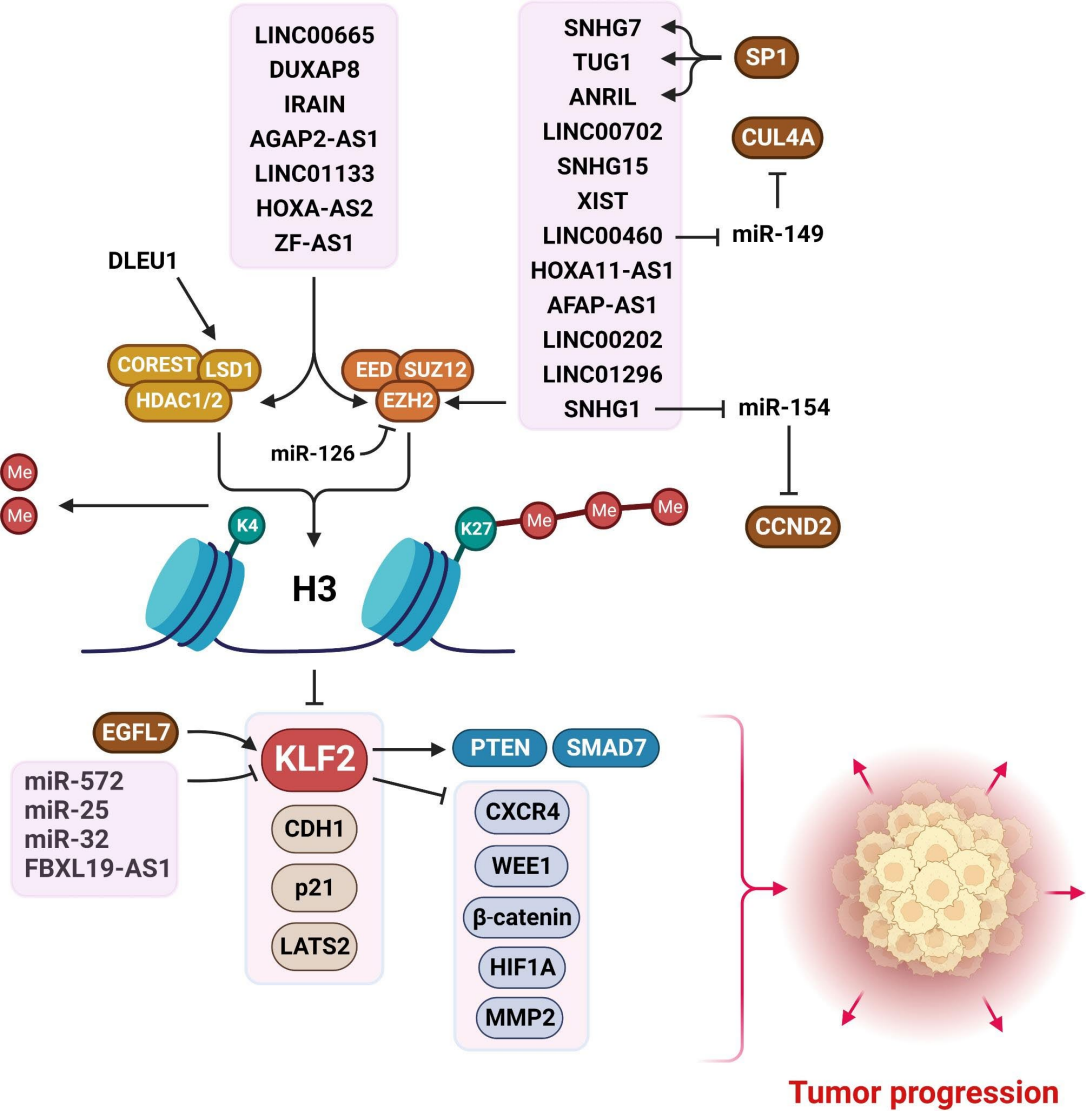
KLF2 is mainly targeted by the oncogenic ncRNAs during tumor progression. (Created with BioRender.com)

Despite significant advances in cancer treatment, it is still considered as one of the main causes of human deaths globally [1, 2]. Surgery, hormone therapy, chemo-radio therapy, and targeted therapy are among the routine cancer treatment options. In some circumstances, the treatment plan may include a variety of therapeutic methods to maximize the therapeutic efficiency. Radiotherapy and surgery are the most effective treatments for non-metastatic and localized tumors but are ineffective in metastatic cancers. As a result, metastasis is the leading cause of cancer death, accounting for more than 90% of all cancer mortalities [3]. Since, anticancer drugs can reach any part of the body through the bloodstream, they are considered as the common treatment options for metastatic tumors [4]. However, chemotherapeutic side effects and multidrug resistance highlight the need for novel and effective targeted therapies based on molecular tumor biology [5, 6]. Such targeted therapies disrupt particular oncogenes and signaling pathways to trigger apoptosis and immune system stimulation with a lower side effects compared with chemotherapeutic modalities [7]. Transcription factors are the main transcriptional regulators [8]. Accordingly, understanding the role of transcription factors, downstream targets, and upstream regulators in tumor cells can help us to develop novel therapeutic approaches to overcome drug resistance [9]. Krüppel-like factors (KLFs) are a family of developmental transcription factors that participate in the modulation of cell growth and differentiation [10, 11]. KLF2 belongs to the KLF protein family that contains Cys2/His2 zinc-finger domains to interact with GC boxes in promoter sequences and exert as transcriptional activators or suppressors [12]. KLF2 can be regulated by ubiquitination, non-coding RNAs, and signaling pathways [13–17]. The polycomb repressive complex 2 (PRC2) belongs to the Polycomb proteins complex that inhibits gene expression through histone modification. PRC2 consists of EZH2, EED, and SUZ12 components. EZH2 catalyzes the H3K27me3 that results in transcriptional inhibition [18]. It has been reported that PRC2 complex has a key role in regulation of KLF2 expression in tumor cells via histone methylation in promoter region [19, 20]. KLF2 functions as a tumor suppressor or oncogene in different tumors [21, 22]. Therefore, in the present review, we discussed the molecular mechanisms of the KLF2 during tumor progression to introduce that as a reliable diagnostic and therapetutic target in cancer patients (Tables 1 and 2) (Fig. 1).

**Table 1:** Oncogenic ncRNAs inhibit the KLF2 by PRC2 recruitment

ncRNA	KLF2 regulation	Tumor Type	Samples	KLF2 Function	Clinical Application	Year	Study
TUG1	KLF2 down regulation	Hepatocellular carcinoma	77T 77 N tissues HepG2, Hep3B, MHCC-97 H cell lines Nude mice	Tumor suppressor	Diagnosis	2015	Huang (15)
ZFAS1	KLF2 down regulation	Gastric cancer	54T 54 N tissues BGC823, SGC7901, MGC803, AGS, HGC27 cell line Nude mice	Tumor suppressor	Diagnosis	2017	Nie (19)
DLEU1	KLF2 down regulation	Gastric cancer	68T 68 N tissues AGS, SGC7901, MGC803, and BGC823 cell lines	Tumor suppressor	Diagnosis	2018	Li (20)
LINC00202	KLF2 down regulation	Gastric cancer	60T 60 N tissues AGS and SGC-7901 cell lines	Tumor suppressor	Diagnosis	2021	Xu (40)
LINC01296	KLF2 down regulation	Esophageal squamous cell carcinoma	78T 78 N tissues EC109, EC9706, and TE-1 cell lines Nude mice	Tumor suppressor	Diagnosis	2018	Wang (41)
AFAP1-AS1	KLF2 down regulation	Gastric cancer	SGC-7901 and AGS cell lines	Tumor suppressor	Diagnosis	2020	Yuan (42)
MiR-32-5p	KLF2 down regulation	Oral squamous cell carcinoma	30T 30 N tissues SCC-9, SCC-15, SCC-25, CAL-27, and Tca-8113 cell lines	Tumor suppressor	Diagnosis	2022	Qin (47)
HOXA11-AS	KLF2 down regulation	Gastric cancer	BGC823, SGC7901 and AGS cell lines Nude mice	Tumor suppressor	Diagnosis	2017	Liu (50)
LINC00460	KLF2 down regulation	Colorectal cancer	60T 60 N tissues HCT116, SW480, HT-29, and Lovo cell lines Nude mice	Tumor suppressor	Diagnosis	2018	Lian (58)
L22NC03-N64E9.1	KLF2 down regulation	Colorectal cancer	50T 50 N tissue DLD-1, Lovo, HT-29, SW480, SW620, and HCT116 cell line	Tumor suppressor	Diagnosis	2017	Lian (59)
SNHG1	KLF2 down regulation	Colorectal cancer	160T 80 N tissues HCT-116, HCT-8, SW-480, SW-620, DLD-1 and HT-29 cell lines Nude mice	Tumor suppressor	Diagnosis	2018	Xu (67)
HOXA-AS2	KLF2 down regulation	Colorectal cancer	69T 69 N tissues HCT116, DLD1, SW480, SW620, HT-29 and LOVO cell lines Nude mice	Tumor suppressor	Diagnosis	2017	Ding (68)
miR-25-3p	KLF2 down regulation	Colorectal cancer	27T 27 N tissues SW480, LS174T, SW620, LOVO, and HCT116 cell lines Nude mice	Tumor suppressor	Diagnosis	2018	Zeng (75)
LINC01133	KLF2 down regulation	Non-small cell lung cancer	68T 68 N tissues PC9, SPC-A1, NCI-H1975, H1299, and A549 cell lines Nude mice	Tumor suppressor	Diagnosis	2016	Zang (83)
miR-572	KLF2 down regulation	Non-small cell lung cancer	46T 46 N tissues A549, H1299, PC-9, H358, and SPC-A1 cell lines	Tumor suppressor	Diagnosis	2022	Sun (85)
AGAP2-AS1	KLF2 down regulation	Non-small cell lung cancer	80T 80 N tissues A549, PC9, SPCA1, H1975 and H1299 cell lines Nude mice	Tumor suppressor	Diagnosis	2016	Li (91)
LINC00511	KLF2 down regulation	Non-small cell lung cancer	57T 57 N tissues A549, PC9 and H460 cell lines	Tumor suppressor	Diagnosis	2019	Zhu (92)
XIST	KLF2 down regulation	Non-small cell lung cancer	53T 53 N tissues A549, SK-MES-1, H1299, 95D, H460, H520, H1975, H157, SK-LU-1, and SPC-A-1 cell lines Nude mice	Tumor suppressor	Diagnosis	2016	Fang (93)
miR-126-5p	KLF2 up regulation	Lung adenocarcinoma	78T 78 N tissues H1975, A549 and H1650 cell lines Nude mice	Tumor suppressor	Diagnosis	2022	Han (94)
FBXL19-AS1	KLF2 down regulation	Hepatocellular cancer	60T 60 N tissues SMMC7721 and HCCLM3 cell lines	Tumor suppressor	Diagnosis	2021	Chen (111)
ANRIL	KLF2 down regulation	Hepatocellular cancer	77T 77 N tissues HepG2, Hep3B, and MHCC-97 H cell lines BALB/c nude mice	Tumor suppressor	Diagnosis	2015	Huang (112)
DUXAP8	KLF2 down regulation	Hepatocellular carcinoma	HepG2 and Hep3 cell lines	Tumor suppressor	Diagnosis	2019	Jiang (113)
IRAIN	KLF2 down regulation	Pancreatic cancer	37T 37 N tissues AsPC-1, BxPC-3, and PANC-1 cell lines	Tumor suppressor	Diagnosis	2016	Lian (128)
SNHG15	KLF2 down regulation	Pancreatic cancer	48T 48 N tissues AsPC-1, BxPC-3 and PANC-1 cell lines Nude mice	Tumor suppressor	Diagnosis	2017	Ma (129)
DUXAP8	KLF2 down regulation	Pancreatic cancer	58T tissues AsPC-1, BxPC-3, and PANC-1 cell lines Athymic mice	Tumor suppressor	Diagnosis	2018	Lian (130)
LINC00702	KLF2 down regulation	Ovarian cancer	36T 36 N tissues ES-2, SKOV-3, A2780, and HEY cell lines	Tumor suppressor	Diagnosis	2019	Wang (137)
SNHG7	KLF2 down regulation	Ovarian cancer	30T 30 N tissues A2780, OCC1, H8710 and SK-OV3 cell lines Nude mice	Tumor suppressor	Diagnosis	2020	Bai (138)
GHET1	KLF2 down regulation	Prostate cancer	30T 30 N tissues LNCap and C4-2 cell lines	Tumor suppressor	Diagnosis	2019	Zhu (143)
LINC00665	KLF2 down regulation	Prostate cancer	50T 50 N tissues PC-3, DU-145, 22RV1, LNCaP cell lines Nude mice	Tumor suppressor	Diagnosis	2021	Xue (144)
SNHG6	KLF2 down regulation	Osteosarcoma	58T 58 N tissues KHOS, MG-63, and U2OS cell lines Nude mice	Tumor suppressor	Diagnosis	2018	Ruan (165)

* Tumor (T) tissues and Normal (N) margins

**Table 2:** KLF2 functions as a tumor suppressor or oncogene by the transcriptional regulation of target genes

KLF2 target gene	Tumor Type	Samples	KLF2 Function	Clinical Application	Year	Study
TGF-β/Smad down regulation	Hepatocellular carcinoma	SMMC-7721, MHCC97H, MHCC97L, and HCCLM3 cell lines	Tumor suppressor	Diagnosis	2020	Li (14)
PTEN/AKT down regulation	Gastric cancer	15T 15 N tissues HGC-27, SNU-1, SGC-7901, NCI-N87, KATOIII, AGS, MKN-28, MKN-45, BGC-823, MGC-803 and GES-1 cell lines Nude mice	Tumor suppressor	Diagnosis and prognosis	2017	Wang (54)
P21 up regulation	Colon cancer	HCT116, mutp53, SW1116, and HEK293A cell lines	Tumor suppressor	Diagnosis	2019	Lu (66)
HIF-1Α/ NOTCH-1 down regulation	Colorectal cancer	SW480 HT29, SW620 and HCT116 cell lines	Tumor suppressor	Diagnosis	2017	Wang (74)
WEE1 down regulation	Ovarian cancer	OV167, OV177, OV202 and OV207 cell lines	Tumor suppressor	Diagnosis	2005	Wang (77)
P21/ P15 up regulation	Non-small cell lung cancer	113T 113 N tissues A549, SPC-A1, SK-MES-1, NCI-H1299, and NCI-H1650 cell lines	Tumor suppressor	Diagnosis and prognosis	2015	Yin (82)
P15/ P21 up regulation	Non-small cell lung cancer	47T 47 N tissues A549, HCC827, SK-MES-1, NCI-H1299 and NCI-H1975 cell lines	Tumor suppressor	Diagnosis and prognosis	2017	Jiang (84)
Glutamine transaminase down regulation	Non-small cell lung cancer	A549, NCI-H1299 cell lines	Tumor suppressor	Diagnosis	2020	Xiao (99)
Hedgehog/Gli1 down regulation	Hepatocellular carcinoma	38T 38 N tissues L02, Chang, 7404 and Huh-7 cell lines Nude mice	Tumor suppressor	Diagnosis	2019	Lin (104)
C-Myc up regulation	Hepatocellular carcinoma	60T 60 N tissues HuH-7 and HepG2 cell lines Nude mice	Oncogene	Diagnosis	2016	Zou (114)
B-Catenin/TCF down regulation	Pancreatic cancer	52T 52 N tissues BXPC3 and Suit2 cell lines	Tumor suppressor	Diagnosis	2016	Zhang (127)
P21 up regulation	Pancreatic cancer	HPAC and SW1990 cell lines Nude mice	Tumor suppressor	Diagnosis	2020	Yuedi (131)
P16, P21, And P27 up regulation and CCND1/ Survivin down regulation	Breast cancer	20T 20 N tissues MCF10A, MCF-7, T47D, SK-BR-3, CAL-51 and MDA-MB-231cell lines	Tumor suppressor	Diagnosis	2022	Zhu (136)
MMP2 down regulation	Prostate cancer	30T 30 N tissues PC-3 and 22Rv1 cell lines	Tumor suppressor	Diagnosis	2019	Wang (142)
IRF4 up regulation	Multiple myeloma	3T tissues RPMI8226, MM.1 S and U266 cell lines	Oncogene	Diagnosis	2016	Ohguchi (154)

* Tumor (T) tissues and Normal (N) margins

### Gastric, esophageal, and oral cancers

SUZ12 has a pivotal role in promotion of tumor cell proliferation and metastasis. Up regulation of SUZ12 has been observed in different types of human cancers [23–25]. There was significant SUZ12 up regulation in gastric cancer (GC) tissues that was associated with distant metastasis, tumor size, stage, and lower survival. SUZ12 induced GC cell proliferation and metastasis by KLF2 and CDH1 down regulations [26]. LSD1 is one of the components of CoREST transcriptional co-suppressor complex by demethylation of H3K4m1/m2 [27, 28]. Several studies demonstrated that LSD1 plays critical roles in cell growth, differentiation, EMT, and invasion [29–31]. Down regulation of LSD1 reduced GC cell proliferation and invasion while promoted apoptosis. LSD1 had an oncogenic role via inhibition of KLF2 through H3K4 demethylation [32]. Long non-coding RNAs (LncRNAs) are involved in X chromosome inactivation, self-renewal, differentiation, and apoptosis [33–35]. Deregulation of lncRNAs was also associated with several cancers by the modulation of gene expression through chromatin remodeling, histone modification, and microRNAs sponging [36, 37]. They are also correlated with tumor cell metastasis and poor prognosis [38, 39]. There was significant DLEU1 up regulation in GC tissues that was linked with the poor prognosis. Downregulation of DLEU1 suppressed the proliferation of GC cells by provoking cell cycle arrest. DLEU1 directly interacted with LSD1 in promoter regions of KLF2, consequently promoting H3K4me2 modification [20]. Suppression of ZFAS1 reduced GC cell growth while promoted apoptosis. ZFAS1 recruited the EZH2 and LSD1 to NDK2 and KLF2 promoters that inhibited their transcription through H3K27me3 and demethylation of H3K4me2. ZFAS1 had a critical role in inhibition of tumor suppressors by EZH2 and LSD1 recruitments in GC cells [19]. There were LINC00202 up regulations in GC tissues and cells. Downregulation of LINC00202 significantly decreased the GC cell proliferation. The KLF2 expression level was affected by the high level of LINC00202 that recruited the EZH2. LINC00202 attenuated the GC progression by KLF2 inhibition [40]. There was significant LINC01296 up regulation in esophageal squamous cell carcinoma (ESCC) tissues compared with normal tissues that was correlated with lymph node metastasis, TNM stage, and poor prognosis. Silencing of LINC01296 decreased ESCC cell proliferation and invasion. LINC01296 down regulated the KLF2 via binding to EZH2 in ESCC cells [41]. There was AFAP1-AS1 up regulation in GC tissues. AFAP1-AS1 induced GC cell proliferation and invasion through KLF2 targeting [42].

Epithelial-to-mesenchymal transition (EMT) is known as a critical biological process in which epithelial cells are altered into mesenchymal cells during particular physiological and pathological contexts to acquire invasive properties [43, 44]. CXCR4 is activated via binding to SDF1, which is a critical oncogene [45]. It has been shown that CXCR4 was significantly correlated with EMT in lung cancer [46]. MiR-32-5p up regulated the CXCR4 through KLF2 targeting, which induced cell proliferation and EMT process in oral squamous cell carcinoma (OSCC) cells [47]. β-catenin is the critical modulator of the Wnt/β-catenin signaling pathway that is involved in tumor progression [48]. Wnt pathway also participates in GC progression via EMT modulation [49]. HOXA11-AS induced GC cell progression by β-catenin up regulation via WDR5 interaction, KLF2 down regulation, and EZH2 mediated P21 inhibition [50]. PI3K/AKT signaling as the main down stream cascade of the growth factor receptors has a key role in tumor progression [51, 52]. PTEN functions as a tumor suppressor by the inhibition of PI3K/AKT [53]. It has been reported that KLF2 was significantly down regulated in GC tissues compared to normal tissues that was associated with overall survival. KLF2 reduced cell migration while promoted apoptosis by the suppression of PTEN/AKT signaling in GC cells. It promoted PTEN expression and inhibited AKT-mTOR signaling. KLF2 also induced apoptosis by regulating p16/CDKN2A and p27/CDKN1B [54].

### Colorectal cancer

CUL4A belongs to the cullin family of proteins that functions as an oncogene by regulation of cell proliferation, differentiation, and apoptosis [55–57]. There was LINC00460 up regulation in colorectal cancer (CRC) tissues that was positively associated with lymph node involvement, stage, and tumor size. Downregulation of LINC00460 reduced CRC cell proliferation while promoted apoptosis. LINC00460 functioned as an oncogene by engaging EZH2 and H3K27me3 to the KLF2 promoter, consequently the inactivation of KLF2. LINC00460 negatively regulated miR-149-5p to up regulate CUL4A. LINC00460 inhibition repressed CRC progression through either EZH2/KLF2 and miR-149-5p/CUL4A pathways [58]. There was L22NC03-N64E9.1 up regulation in CRC tissues that was correlated with CRC progression. L22NC03-N64E9.1 induced CRC cell proliferation through down regulation of KLF2 via interacting with EZH2 [59].

The p53 is a tumor suppressor that plays an important role in mediating cell cycle arrest, apoptosis, and genomic stability [60]. It inhibits tumor progression via mediating transcription of different downstream target genes which are participated in apoptosis and cell-cycle arrest [61]. Several studies found that simvastatin had therapeutic influence on several types of cancers by NF-kB, AKT, JNK, and CASP3/Bcl-2/cIAP mediated apoptosis [62–65]. Simvastatin remarkably up regulated KLF2 in p53-muted colon cancer cells. KLF2 was demonstrated to intervene in the anti-proliferative impact and anti-metastasis consequence of simvastatin on mutp53 colon cancer cells. Anti-proliferative effects of KLF2 were revealed by p21 up regulation in mutp53 cancer cells [66].

CDKN2B belongs to the cyclin-associated kinase inhibitors that may form a complex with CDK4 or CDK6 and inhibits the activation of the cyclin-dependent kinase to suppress cell cycle. There was significant SNHG1 up regulation in CRC tissues that was associated with poor prognosis. SNHG1 up regulated the CCND2 through the miR-154-5p sponging. SNHG1 interacted with EZH2 for a PRC2-associated down regulation of KLF2 and CDKN2B [67]. HOXA-AS2 induced CRC cell proliferation by promotion of cell proliferation while inhibition of apoptosis. HOXA-AS2 epigenetically suppressed the p21 and KLF2 transcription via interacting with EZH2 and LSD1 [68].

HIF-1α is an important modulator of hypoxic response in cancer cells [69]. Accumulating evidence revealed that hypoxia plays a critical role in tumor progression, angiogenesis, distant metastasis, and cancer therapy [70, 71]. It has been demonstrated that HIF-1α can interact with the Notch target gene to regulate its signaling in cancer stem cells [72]. Notch-1 induces tumorigenesis in CRC and preserves cells from apoptosis [73]. KLF2 repressed CRC cell growth through suppressing the HIF-1α/ Notch-1 axis [74]. Exosomal miR-25-3p intervened in the construction of a pre-metastatic niche in nude mice through promotion of the vascular permeability and subsequent CRC metastasis. MiR-25-3p targeted the KLF2 and KLF4 in HUVECs that resulted in ZO-1, occludin, and Claudin5 down regulations while VEGFR2 up regulation [75].

### Lung cancer

KLF2 inhibits the leukemia cell growth by p21 up regulation [76], while down regulates the Wee1 to promote apoptosis [77]. WW domain-containing protein 1 (WWP1) induces the ubiquitination and degradation of KLF2 [78]. Smurf1 plays important roles in regulating cell polarity and tumor progression via mediating BMP-Smad, RhoA signaling pathways [79, 80]. WWP1 and Smurf1/2 have been also demonstrated to mediate Smads degradation in TGF-b signaling pathway [81]. Smurf1 as the HECT-type ubiquitin ligase might promote the KLF2 degradation in lung tumor cells [13]. There were significant KLF2 down regulations in non small-cell lung cancer (NSCLC) tissues that was correlated with tumor size, tumor stage, lymphatic metastasis, and survival. KLF2 repressed NSCLC cell growth via p21 and p15 targeting [82]. There was LINC01133 up regulation in NSCLC cells that was correlated with poor prognosis. LINC01133 had an oncogenic role in NSCLC cells through associating with EZH2 and LSD1, and KLF2, P21, and CDH1 down regulations. LINC01133 promoted EMT via CDH1 down regulation in NSCLC cells [83]. It has been indicated that KLF2 was notably downregulated in NSCLC tissue samples that was correlated with NSCLC lymph node metastasis and advanced TNM stage. KLF2 remarkably inhibited tumor cell viability while induced apoptosis through the expression of p15 and p21 in NSCLC cells [84]. There was miR-572 up regulation in NSCLC samples that was significantly correlated with metastasis and prognosis. MiR-572 promoted NSCLC cell proliferation and migration via KLF2 targeting [85].

EZH2 is a catalytic subunit of PRC2 that has a histone methyltransferase function to mediate the H3K27me3 tails of different target genes [86]. LSD1 is a histone demethylase as the core subunit of the REST suppressor which particularly demethylases H3K4me1/2 [87]. LncRNAs can modulate the transcription of target genes by interacting with PRC2 [88]. LATS2 belongs to the LATS family of protein kinases that is involved in spindle construction and genome integrity [89, 90]. It has been documented that there was AGAP2-AS1 up regulation in NSCLC tissues that was correlated with poor prognosis. AGAP2-AS1 acted as an oncogene in NSCLC cells through the LATS2 and KLF2 down regulations. AGAP2-AS1 recruited the EZH2 and LSD1 to down regulate the LATS2 and KLF2 in NSCLC cells [91]. LINC00511 induced the NSCLC progression via LATS2 and KLF2 down regulations followning the recruitment of EZH2 and LSD1 to their promoter sequences, respectively [92]. XIST mediates cell proliferation and invasion via epigenetically inhibiting KLF2 in NSCLC cells. There was XIST up regulation in NSCLC tissues that was associated with poor prognosis and poor overall survival. KLF2 acts as tumor suppressor in NSCLC cells and its’ expression could be repressed through XIST via recruiting EZH2 to its promoter region [93]. MiR-126-5p down regulation was found in lung adenocarcinoma tissues that was correlated with poor prognosis. MiR-126-5p suppressed EZH2 to increase the expression level of KLF2 and decreased BIRC5 expression, that inhibited lung tumor cell proliferation, migration while increased radiosensitivity and apoptosis [94].

The ATP production by glycolysis is lower than oxidative phosphorylation that can be substituted by the higher glucose absorption in tumor cells. Glycolysis also supplies many nutrients to maintain the tumor cell proliferation [95, 96]. Tumor cells have a high level of the glutamine consumption to prepare their required energy for the cell proliferation and growth [97, 98]. It was shown that KLF2 significantly reduced the NSCLC cell proliferation through the reduced glutamine consumption following the glutamine transaminase down regulation [99].

### Hepatocellular cancer

Hedgehog (Hh) signaling pathway participates in the promotion of tumor cell growth and metastasis [100]. Sonic (Shh), Desert (Dhh), and Indian (Ihh) encode secretory proteins that act as Hh ligands [101]. The secreted Hh ligand associates with Hip1, Patched 2 (Ptch2), and Ptch1 as transmembrane receptors through dissemination [102]. The Ptch receptor inhibits the effect of Smo in ligand loss. Activation of Smo may induce Gli1 transcription factor [103]. There was KLF2 down regulation in hepatocellular cancer (HCC) tissue that suppressed the cell growth and metastasis by repressing the Hedgehog/Gli1 signaling cascade. KLF2 competed with Gli1 to interact with HDAC1 to inhibit the Hedgehog signal [104].

TGF-β belongs to the TGF-β cytokine family that contains activin, nodal, and bone morphogenetic proteins [105]. TGF-β signaling is activated by TGF-β ligand that promotes Smad2/3 through phosphorylation. Then smad2/3/4 oligomeric complex enters into the nucleus to regulate the TGF-β target genes [106–108]. KLF2 has been found to reduce TGFβ/Smad signaling in endothelial cells through Smad7 up regulation [109, 110]. TGF-β promoted the expression of KLF2 in numerous HCC cells. KLF2 suppressed the TGF-β/Smad pathway by provoking the transcriptional activity of Smad3 and Smad4 [14].

FBXL19-AS1 promoted the HCC cell proliferation while inhibited apoptosis via KLF2 down regulation [111]. There was ANRIL up regulation in HCC tissues that was associated with tumor size and stage. It may modulate cell growth by epigenetic inhibition of KLF2 via interacting with PRC2. The expression of ANRIL could also be regulated by SP1. SP1-mediated ANRIL expression modulated the KLF2 expression. ANRIL suppressed KLF2 transcription through cooperating with EZH2 and SUZ12 in HCC cells and recruitment of PRC2 to the KLF2 promoter [112]. DUXAP8 was considerably up regulated in HCC that was correlated with poor prognosis. DUXAP8 induced HCC cell growth by KLF2 down regulation [113]. There was TUG1 up regulation in HCC tissues that was associated with tumor size and BCLC stage. The high expression level of TUG1 was promoted through SP1 and mediated HCC cell growth by epigenetically inhibiting KLF2 via interacting with PRC2 [15]. KLF2 was significantly up regulated in HCC tissues compared to surrounding normal liver tissues. KLF2 induced HCC cell proliferation through c-MYC targeting [114].

### Pancreatic cancer

CDK protein family has critical roles in cell cycle and gene expression regulation through interacting with transcription factors to mediate RNA polymerase II activity [115–117]. CDK8 as a part of the mediator complex, which contains cyclin C, MED12, and MED13, modulates transcription [118–120]. CDK8 induces the β-catenin expression in pancreatic cancer that enters into the nucleus to mediate the activation of angiogenesis-promoting transcription factors [121–123]. KLF2 is a critical downstream target of β-catenin that is involved in transcriptional regulation of several target genes [16, 17]. KLF2 was also known as a critical transcriptional modulator of endothelial inflammation, that could suppress VEGF-associated angiogenesis and tissue edema, and its upregulation may elevate the expression level of Semaphorin-3 F (SEMA3F) [124, 125]. The high expression level of CDK8 in pancreatic cancer was considerably associated with poor prognosis. KLF2 inhibited cancer cell angiogenesis. CDK8 was critical for tumor vessel progression in pancreatic carcinoma through the β-catenin-KLF2 axis [126]. KLF2 inhibited the growth and metastasis of pancreatic cancer cells by interaction with b-catenin that suppressed the activity of b-catenin/TCF complex [127].

P15 is a CDK inhibitor that functions through inhibition of CDK activation by CCND resulting in cell cycle G1 arrest. There was significant IRAIN up regulation in pancreatic cancer tissues that was associated with larger tumor sizes, higher TNM stages, and lymph node metastasis. IRAIN induced cell proliferation directly through interacting with EZH2 and LSD1 complexes and suppressing KLF2 and P15 in pancreatic cancer cells [128]. SNHG15 induced pancreatic cancer cell proliferation by repressing P15 and KLF2 expression via EZH2-related H3K27me3 [129]. There was significant DUXAP8 up regulation in pancreatic cancer tissues that was correlated with the larger size of the tumor, advanced clinical stage, and shorter survival rate. DUXAP8 promoted the cell proliferation and tumor progression in pancreatic cancer through p21 and KLF2 down regulations following interaction with EZH2 and LSD1 [130]. KLF2 also induced pancreatic tumor cell senescence through cooperating with FOXO4 and promoting the expression of p21 [131].

### Breast, ovarian, and prostate cancers

Wee1 is a tyrosine kinase that regulates cell cycle progression. M-phasepromoting factor (MPF), which is a member of the CDC2 and cyclin B complex, could mediate the G2/M transition through cell cycle. MPF is required for mitosis and is also necessary for DNA damage-associated apoptosis. Wee1 negatively modulates the MPF complex via CDC2 phosphorylation that leads to mitosis deregulation and resistance to apoptosis [132–134]. KLF2 recruited the SP1/CPBP to down regulate the WEE1 that sensitized ovarian tumor cells toward the DNA damage mediated apoptosis [77]. It has been suggested that KLF2 can be introduced as a promising target to increase the sensitivity of breast cancer (BCa) to cisplatin via down regulation of WEE1 [135]. KLF2 promoted cell apoptosis while inhibited cell proliferation through p16, p21, and p27 up regulations and CCND1 and survivin down regulations in breast tumor cells [136]. Silencing of LINC00702 decreased the ovarian cancer (OC) cell proliferation. It facilitated the progression of OC through binding to EZH2 to suppress the transcription of KLF2 [137]. SNHG7 inhibition reduced the OC cell growth and invasive. SP1 up regulated the SNHG7 that interacted with EZH2 to inhibit KLF2 expression in ovarian tumor cells [138].

Matrix metalloproteinases (MMPs) are a multifunctional family of zinc-dependent endopeptidases that has a critical role in the degradation of the extracellular matrix (ECM). These extracellular molecules are secreted via cells that supply structural and biochemical associates with normal physiological cells. MMP2 has a critical role in tumor cell migration due to the degradation of collagens [139–141]. KLF2 was considerably down regulated in prostate cancer (PCa) tissues in comparison with normal margins. It suppressed the prostate tumor cell invasion through MMP2 inhibition [142]. KLF2 inhibits CRC cell proliferation by promoting HIF-1α/Notch-1 signal pathway [74]. There was GHET1 up regulation in PCa tissues that was negatively linked to KLF2 expression. GHET1 promoted the PCa progression through decreasing the level of KLF2 expression. Accumulating evidences demonstrated that HIF-1α and Notch-1 were also considerably down regulated through GHET1 inhibition in prostate tumor cells [143]. LINC00665 was significantly up regulated in PCa tissues and cell lines. LINC00665 down regulation reduced the PCa cell proliferation and migration. It facilitated the malignant progression of PCa by epigenetically suppressing the expression level of KLF2 via interaction with EZH2 and LSD1 [144].

### Myeloma and osteosarcoma

Histone methylation is one of the main regulators of chromatin remodeling and gene expression that is required for several biological activities such as cell proliferation, DNA damage, and stress response [145, 146]. KDM3A belongs to the Jumonji histone demethylases family that acts as a coactivator for the androgen receptor and mediates the elimination of H3K9me1 and H3K9me2 [147]. It has a critical role as a modulator of spermatogenesis, self-renewal, metabolic gene expression, and sex resolvation [147–150]. IRF4 belongs to the interferon regulatory family of transcription factors that has a crucial role in mediating the plasma cell differentiation [151–153]. Silencing of KDM3A induced apoptosis in myeloma cells through KLF2 and IRF4 up regulations by H3K9 elimination. Down regulation of KLF2 promoted apoptosis and that KLF2 positively regulated the IRF4 promoter [154].

The epidermal growth factor-like protein-7 (EGFL7) stimulates endothelial cell survival, migration, and differentiation [155, 156]. Deregulation of EGFL7 has been frequently observed in multiple types of solid tumors and acute myeloid leukemia [157, 158]. Multiple myeloma (MM) cells can evade drug treatment via integrin-mediated cellular adhesion. ITGB3 promotes MM cell proliferation, protease secretion, and invasion [159–161]. EGFL7 is involved in angiogenesis by interaction with ITGB3 and Notch receptors [162]. EGFL7 induced MM growth through ITGB3 and KLF2 up regulations [163].

KLF2 functions as a tumor suppressor by PCNA and CCND1 down regulations while p21 up regulation [164]. It has been documented that SNHG6 was up regulated in osteosarcoma tissues that was correlated with tumor grade and shorter overall survival. Downregulation of SNHG6 repressed cell proliferation while increased apoptosis. There was a negative association between p21, KLF2, and SNHG6 and a positive association between CCND1 and SNHG6. SNHG6 facilitated the osteosarcoma cell proliferation via p21 and KLF2 modulations [165].

## Conclusions

It has been reported that the KLF2 has mainly a tumor suppressor function that can be suppressed by the oncogenic ncRNAs following the PRC2 recruitment. On the other hand, ncRNAs can promote the tumor cell growth and proliferation by PRC2 mediated KLF2 targeting. Therefore, KLF2 and its ncRNA regulators can be introduced as appropriate therapeutic and diagnostic targets in cancer patients. Considering that the PRC2 complex as an KLF2 inhibitor has an oncogenic role, PRC2 inhibitors can indirectly inhibit the tumor growth and progression by KLF2 activation. On the other hand, it has been shown that lncRNAs promote PRC2-mediated KLF2 down regulation in tumor cells. Therefore, inhibition of lncRNAs/PRC2 axis can up regulate the KLF2 to reduce tumor progression. Besides the therapeutic importance, lncRNAs/PRC2/KLF2 axis can also be used as a diagnostic/prognostic marker in cancer patients. However, due to the pivotal role of KLF2 and PRC2 in normal cellular processes, targeted therapy against the PRC2/KLF2 axis results in side effects in normal cells and tissues. Therefore, it is required to use the novel methods to deliver the inhibitors of PRC2/KLF2 axis locally and specifically to the tumor tissue in order to reduce the side effects as much as possible. Indeed, further animal studies and clinical trials are needed to be able to use the lncRNAs/PRC2/KLF2 axis for diagnostic and therapeutic purposes in cancer patients.

## Data Availability

The datasets used and/or analyzed during the current study are available from the corresponding author on reasonable request.

## List of Abbreviations

BCa: Breast cancer
CRC: Colorectal cancer
Dhh: Desert
EGFL7: Epidermal growth factor-like protein-7
EMT: Epithelial-to-mesenchymal transition
ESCC: Esophageal squamous cell carcinoma
ECM: Extracellular matrix
GC: Gastric cancer
Hh: Hedgehog
HCC: Hepatocellular cancer
Ihh: Indian
KLF2: Krüppel-like factors 2
LncRNAs: Long non-coding RNAs
MMPs: Matrix metalloproteinases
MPF: M-phasepromoting factor
MM: Multiple myeloma
ncRNAs: Non-coding RNAs
NSCLC: Non small-cell lung cancer
OSCC: Oral squamous cell carcinoma
OC: Ovarian cancer
Ptch2: Patched 2
PRC2: Polycomb repressive complex 2
PCa: Prostate cancer
SEMA3F: Semaphorin-3 F
Shh: Sonic
WWP1: WW domain-containing protein 1
